# Supplementary material for: Primary determinants of water yield services in arid NW China: An empirical analysis of Gansu Province
Source: PLoS One. 2025 Aug 6;20(8):e0329580. doi: 10.1371/journal.pone.0329580 (PMC12327602; doi:10.1371/journal.pone.0329580)
Supplement: S1 Table — (DOCX) [file pone.0329580.s001.docx]

Table 1 The Biophysical Parameters Table Utilized in the InVEST Water yield Model

| Lucode | LULC_desc | LULC_veg | Kc | Root_depth(mm) |
| --- | --- | --- | --- | --- |
| 11 | Paddy feld | 1 | 0.70 | 2100 |
| 12 | Dry land | 1 | 0.65 | 2000 |
| 21 | Forestland | 1 | 1.0 | 5200 |
| 22 | Shrub land | 1 | 0.95 | 5200 |
| 23 | Wood land | 1 | 0.90 | 4800 |
| 24 | Other forest | 1 | 0.85 | 2500 |
| 31 | High grassland | 1 | 0.80 | 2500 |
| 32 | Mid grassland | 1 | 0.65 | 2300 |
| 33 | Low grassland | 1 | 0.60 | 2000 |
| 41 | Canal | 0 | 1.0 | 100 |
| 42 | Lake | 0 | 1.0 | 100 |
| 43 | Beaches | 0 | 1.0 | 100 |
| 44 | Snow | 0 | 0.5 | 100 |
| 45 | Beaches | 0 | 1 | 1000 |
| 46 | Reservoir | 0 | 1 | 100 |
| 51 | Urban | 0 | 0.3 | 100 |
| 52 | Rural settlement | 0 | 0.2 | 100 |
| 53 | Other built-up land | 0 | 0.2 | 100 |
| 61 | Sand | 0 | 0.2 | 300 |
| 62 | Gobi Desert | 0 | 0.1 | 300 |
| 63 | Saline alkali land | 0 | 1 | 300 |
| 64 | Swamp land | 0 | 1 | 300 |
| 65 | Barren land | 0 | 0.2 | 300 |
| 66 | Bare rocky land | 0 | 0.2 | 300 |

Lucode: A distinctive integer assigned to each Land Use and Land Cover (LULC) class. LULC_desc: The descriptive name of each LULC category pertaining to land use and land cover. LULC_veg: Vegetated land use types, specifically croplands, forests, and grasslands, are designated a value of 1, while other land use types receive a value of 0. Kc: Represents the plant transpiration coefficient specific to each LULC category. Root_depth: Denotes the maximum root depth for vegetated land use classes, measured in millimeters. Note: Cropland encompasses codes 11 and 12. Forests include codes 21, 22, 23, 24. Grassland consists of codes 31, 32, 33. Waters span codes 41 through 46. Built-up land covers codes 51, 52, 53. Unused land comprises codes 61 through 66.
