# Supplementary material for: Primary determinants of water yield services in arid NW China: An empirical analysis of Gansu Province
Source: PLoS One. 2025 Aug 6;20(8):e0329580. doi: 10.1371/journal.pone.0329580 (PMC12327602; doi:10.1371/journal.pone.0329580)
Supplement: Attachment — Submitted filename: Response to Reviewers.docx [file pone.0329580.s004.docx]

**Dear Editors and Reviewers:**

Thank you for your proposed comments on our manuscript entitled "Primary Determinants of Water Yield Services in Arid NW China: An Empirical Analysis of Gansu Province" (Manuscript ID: PONE-D-25-00782). These comments are all valuable and very helpful for revising and improving our paper. We have carefully revised the manuscript based on the reviewers' comments and hope that the revised manuscript meets the requirements.

The main corrections in the paper and the responds to the reviewers' comments are as following:

**Comment 1:** Thank you for updating your data availability statement, You note that your data are available within theSupporting Information files, but no such files have been included with your submission. At this time we askthat you please upload your minimal data set as a Supporting Information file, or to a public repository such asFigshare or Dryad.

**Response 1:** Thank you for your thorough evaluation and constructive feedback. We sincerely apologize for the oversight in not including the Supporting Information files with our initial submission.

All relevant data are within the paper and its Supporting Information files. In response to your request, we have now uploaded the minimal data set as a Supporting Information file, which includes [briefly describe key data files, e.g., "raw experimental measurements, processed datasets, and statistical outputs"]. These files are integral to replicating our analyses and verifying the conclusions presented in the manuscript.

Should the file size or platform limitations affect accessibility, we will promptly deposit the datasets in a public repository such as Figshare or Dryad and provide the corresponding DOI link in the final version.

We deeply appreciate your guidance in ensuring full compliance with data transparency standards. Please do not hesitate to contact us if further adjustments are needed.
