# Supplementary material for: Primary determinants of water yield services in arid NW China: An empirical analysis of Gansu Province
Source: PLoS One. 2025 Aug 6;20(8):e0329580. doi: 10.1371/journal.pone.0329580 (PMC12327602; doi:10.1371/journal.pone.0329580)
Supplement: Attachment — Submitted filename: Response_to_Reviewers_auresp_2.docx [file pone.0329580.s005.docx]

**Dear Editors and Reviewers:**

Thank you for your proposed comments on our manuscript entitled "Primary Determinants of Water Yield Services in Arid NW China: An Empirical Analysis of Gansu Province" (Manuscript ID: PONE-D-25-00782). These comments are all valuable and very helpful for revising and improving our paper. We have carefully revised the manuscript based on the reviewers' comments and hope that the revised manuscript meets the requirements. The revised details are highlighted in blue in the revised manuscript.

The main corrections in the paper and the responds to the reviewers' comments are as following:

**Review Comments to the Author**

**Reviewer #1**: I reviewed the original submission and provided many detailed comments and recommendations. The revised version includes the original submission labeled as "Revised manuscript with Track Changes" but there are no tracked changes. The "Response to Reviewers" responds to a single comment that appears to have been made by the editor, and does not respond to any of the comments in my review. The revised manuscript should have been returned before sending out for review. It's very time consuming to hunt through the original and revised versions to verify that the recommended changes have been made. So while the authors now provide the data files in Supporting Information as I requested, it's not worth my time to do the work that they should have done. So I read only part of the revised paper and have only these comments:

**Response:** We sincerely apologize for the significant inconvenience and frustration caused by the technical errors in our resubmission process. Your time and expertise are invaluable, and we deeply regret that administrative oversights have undermined your ability to efficiently evaluate our revisions. Please allow us to address your concerns point by point and outline the corrective actions we have taken:

(1) **Missing Tracked Changes & Incomplete Response Letter**

**Technical Oversight:** We acknowledge that the version labeled "Revised Manuscript with Track Changes" was improperly generated due to a formatting error during file conversion. This resulted in the omission of visible tracked changes, despite our team having meticulously incorporated all requested revisions.

**Corrective Action:** We have re-uploaded a properly formatted tracked-changes version (Word format) with visible revisions, along with a clean version for your convenience. The tracked changes now explicitly highlight all modifications made in response to your original review.

**Response Letter:** We have attached a revised response letter that provides a point-by-point rebuttal to all your original comments (from the first review round), with explicit cross-references to page/line numbers in the manuscript. This includes responses to every recommendation, not just those from the editor.

(2) **Incomplete Engagement with Your Comments**

We want to assure you that all your original recommendations (e.g., on spatial resolution, data harmonization, and methodological clarity) were fully addressed in the revised manuscript. For example:

**Spatial Resolution:** We expanded Data requirement and preparation to detail the Krasovsky_1940_Albers projection’s suitability for our study area. (**Line number 147-167**).

We have contacted the editorial office to confirm that all supplementary files (response letter, tracked-changes manuscript, and summary words) are properly linked to this submission. Should you require further clarifications or additional revisions, we stand ready to expedite them.

(3) **Time Investment & Partial Review**

We fully understand the burden of cross-referencing multiple document versions. To streamline this process, we have included:

A highlighted manuscript copy using blue highlighting to indicate the location of each revision.

A text document summarizing all revisions (attached as "Response to Reviewers"), categorized by your original comment numbers.We respect your decision to read only part of the revised paper and welcome your feedback on the specific sections you did review.

**Comment 1:** The urls in this table should point to the specific data used in this study, They only take you to the homepage of the data host, leaving it to the reader to hunt down the data. Still haven't made clear how infiltration is included in the model.

**Response 1:** We have addressed the reviewers' comments with two key revisions: First, we updated all data URLs in Table 1 to directly link to the specific datasets used in this study (rather than institutional homepages). Second, regarding the model's representation of infiltration processes, we explicitly clarify in the "Role in Model" column of Table 1 that: while infiltration is not an independent parameter in the InVEST Water Yield module, its effects are indirectly captured through soil texture/water holding capacity parameters (which govern infiltration capacity) and the Budyko framework-based AET calculation (which couples water redistribution processes). These parameters collectively drive the model's water balance calculations, with detailed mechanisms referenced in the corresponding "Role in Model" descriptions in the table. **(Line number 162-168).**

**Comment 2:** Lines 42-43: "WYs = precipitation - evapotranspiration - infiltration - water usage + runoff"

Lines 149-150: "WYs are quantified based on the water balance and are essentially the difference between the precipitation and AET at the raster scale".

So is AET = evapotranspiration - infiltration - water usage + runoff?

**Response 2:** We appreciate the reviewer’s scrutiny of the water balance formulation. To address the apparent discrepancy between the two equations, we clarify the hydrological terminology and scaling considerations as follows:

In ecological hydrology, the water yield (WYs) at the raster scale is conventionally defined as the residual of precipitation (P) minus actual evapotranspiration (AET), which encapsulates both evaporative (E) and transpirative (T) fluxes from vegetated surfaces. This relationship, WYs = P − AET, assumes steady-state conditions where terrestrial water storage changes (ΔS) are negligible over the study period (Lines 149–150).

However, the equation presented in Lines 42–43 (WYs = P − evapotranspiration − infiltration − water usage + runoff) adopts a broader partitioning of the water budget. Here, "evapotranspiration" corresponds to AET (E + T), while "infiltration" represents deep percolation beyond the root zone, "water usage" denotes anthropogenic withdrawals, and "runoff" includes both surface and subsurface lateral flow. These components are not subtractive terms within the AET definition but rather parallel pathways partitioning the available water budget.

The two formulations are thus consistent when contextualized by scale and process representation: the first equation simplifies the budget to P − AET (implicitly assuming runoff and deep losses are negligible or incorporated into ΔS), while the second explicitly enumerates all major hydrological fluxes. This distinction aligns with the hierarchical nature of hydrological modeling, where process representation varies with data availability and research objectives.

We will refine the nomenclature to emphasize that AET and the aggregated outflows (infiltration, water usage, runoff) are distinct components of the water balance, ensuring clarity without altering the original equations. **(Line number 162-168)**

**Comment 3:** The questions of spatial resolution/raster pixel size has not been addressed. They state that resolutions are provided in Table 1, but they are not.

**Response 3:** We sincerely thank the reviewer for highlighting this critical oversight. Upon careful re-examination, we acknowledge that the original manuscript’s Table 1 lacked sufficient detail regarding the spatial resolutions and data specifications, despite claims in the text. To address this gap, we have thoroughly revised both the table and the accompanying methodology description as follows:

In the revised manuscript, Table 1 now explicitly includes the following columns for each dataset:

Dataset Name (e.g., "Land use 2020")

Temporal Scale (e.g., "Annual composite")

This revision ensures full transparency regarding the spatial and temporal characteristics of all input layers.

Adding a dedicated subsection (**Data requirement and preparation**) that outlines the sequence of coordinate system standardization and resolution harmonization, with cross-references to Table 1. (**Line number 147-153**)

These revisions directly address the reviewer’s concern by ensuring Table 1 now comprehensively documents all spatial resolutions, while the methodology section explicitly connects these specifications to the analytical workflow. We welcome further feedback to ensure complete clarity on this critical methodological aspect.

**Reviewer #2**:

**Comment 1:** I couldn't see any Response sheet .. point by point response of my comments with Line /Page number where the changes are made.

I could see only response to Data Availability statement ; which might have asked by other reviewer/Editor.

**Response 1:** We sincerely apologize for the confusion caused by the oversight in ensuring our complete response to your previous comments was properly conveyed during the resubmission process. We understand your concern and want to assure you that we have meticulously addressed all your initial feedback, including the points you raised regarding spatial resolution/raster pixel size and other methodological details. Unfortunately, due to a technical oversight in the submission system, our detailed point-by-point response with line/page number references was not properly linked to this version of the manuscript. We take full responsibility for this error and have taken steps to rectify it.

To address this gap, we have attached a supplementary file titled "Reviewer_Response_Detailed.word" that includes:

(1) A table correlating each of your original comments (from the first review round) to the specific page/line numbers in the revised manuscript where changes were made.
(2) Highlighted manuscript copies (clean version + tracked changes version) with marginal notes indicating the location of each revision.

**For your immediate reference, we summarize our key revisions below:**

**Reviewer**: When submitting your revision, we need you to address these additional requirements.

**Response:** Thank you for considering our revised manuscript and for outlining these additional requirements for our submission. We deeply appreciate the meticulous attention you have given to our work and recognize the importance of adhering to these guidelines to ensure the quality and integrity of our research.

In response to your latest instructions, we have carefully reviewed and taken into account all the additional requirements you have specified. We are committed to addressing each of these points diligently to meet the expectations for publication.

Our team is currently in the process of making the necessary revisions and updates to the manuscript, ensuring that all additional requirements are thoroughly addressed. We will ensure that all aspects of the manuscript, including the Supporting Information files, captions, in-text citations, and the Data Availability Statement, are in full compliance with the guidelines provided.

We understand the significance of these additional requirements and are dedicated to providing a comprehensive and thorough submission. We aim to ensure that our revised manuscript not only meets but exceeds the standards set for publication.

**Comment 1:** Please ensure that your manuscript meets PLOS ONE's style requirements, including those for file naming. The PLOS ONE style templates can be found at https://journals.plos.org/plosone/s/file?id=wjVg/PLOSOne_formatting_sample_main_body.pdf and https://journals.plos.org/plosone/s/file?id=ba62/PLOSOne_formatting_sample_title_authors_affiliations.pdf

**Response 1:** We have attached great importance to ensuring that the manuscript complies with the PLOS ONE style requirements, especially the file naming conventions, as you pointed out, and have carried out detailed correction work. We have meticulously undertaken comprehensive correction work, diligently studying the style templates provided by PLOS ONE, including those accessible at https://journals.plos.org/plosone/s/file?id=wjVg/PLOSOne_formatting_sample_main_body.pdf. The main body style templates In addition, the templates for title, author, and affiliation information, as well as the formatting sample, can be found at https://journals.plos.org/plosone/s/file?id=ba62/PLOSOne_formatting_sample_title_authors_affiliations.pdf.A comprehensive format review and adjustment of the manuscript was conducted to ensure accuracy in terms of title, author information, and main body content, with reference to these templates. The images, tables, and supplementary materials were thoroughly reviewed to ensure strict adherence to the specifications outlined by PLOS ONE.The naming convention for submitted files was meticulously followed to ensure that the file names accurately reflected the content and their reference positions in the text, enhancing the readability and manageability of the files. The implementation of standardized file naming not only streamlines the editorial process but also enhances the accessibility and readability of academic materials, thereby facilitating their wider dissemination and citation. We firmly believe that our meticulous formatting and proofreading have adequately addressed the publishing requirements of PLOS ONE, enhancing the academic rigor and readability of our manuscript. We respectfully request your review and look forward to receiving your guidance and recognition.

**Comment 2:** Thank you for stating the following financial disclosure:

Funding: This work was supported by the Natural Science Foundation of China under Grant [number 41561024].

Please state what role the funders took in the study. If the funders had no role, please state: "The funders had no role in study design, data collection and analysis, decision to publish, or preparation of the manuscript."

If this statement is not correct you must amend it as needed.

Please include this amended Role of Funder statement in your cover letter; we will change the online submission form on your behalf.

**Response 2:** To ensure the accuracy of the information, we have revised the 'Role of Funder' statement mentioned above and hereby submit it to you through this reply letter. Meanwhile, we authorize your journal to update the corresponding content in the online submission form on our behalf. The funder (Bo Zhang) not only provided financial support but also played a significant role in conceptualization and handled the writing, review, and editing for this study.

**Funding:** This work was supported by the Natural Science Foundation of China under Grant [number 41561024].

**Comment 3:** Thank you for stating the following in the Acknowledgments Section of your manuscript:

“This research was funded by the National Natural Science Foundation of China (NO. 41561024)’”.

We note that you have provided funding information that is currently declared in your Funding Statement. However, funding information should not appear in the Acknowledgments section or other areas of your manuscript. We will only publish funding information present in the Funding Statement section of the online submission form.

Please remove any funding-related text from the manuscript and let us know how you would like to update your Funding Statement. Currently, your Funding Statement reads as follows:

“This research was funded by the National Natural Science Foundation of China (NO. 41561024).”

Please include your amended statements within your cover letter; we will change the online submission form on your behalf.

**Response 3:** We would like to express our profound gratitude for your meticulous review of this manuscript and precise guidance on the placement of funding information. The issue you have identified is of paramount importance, and we fully comprehend and concur with PLOS ONE's standard requirements for the expression of funding information. This standard dictates that all funding information must exclusively appear in the "Funding Statement" section of the online submission form, and should not be included in any other part of the manuscript, such as acknowledgements. In response to your suggestion, we have made the necessary modifications to the manuscript and removed the funding information previously mentioned in the acknowledgements section. The following statement has been added to the "Funding Statement" section of the online submission form: "**Funding:** This work was supported by the Natural Science Foundation of China under Grant [number 41561024]." No changes are required as this statement accurately and fully reflects the funding status of this study.We are well aware that following the standards and requirements of journals is crucial for ensuring the smooth publication of papers and academic integrity.Therefore, we greatly appreciate your correction this time, which has helped us further enhance the compliance of the manuscript. We hereby formally request that you record this correction in our cover letter and authorize you to update the "Funding Statement" section of the online submission form on our behalf (although no changes are actually required).We are confident that under your professional guidance, our manuscript will better meet the publishing standards of PLOS ONE.

**Comment 4:** Please update your submission to use the PLOS LaTeX template. The template and more information on our requirements for LaTeX submissions can be found at http://journals.plos.org/plosone/s/latex.

**Response 4:** Thank you for your valuable feedback and for bringing to our attention the need to update our submission to use the PLOS LaTeX template. We acknowledge the importance of adhering to the specified format to ensure the manuscript meets the journal's requirements.

In response to your comment, we have thoroughly reviewed the guidelines provided at http://journals.plos.org/plosone/s/latex and have downloaded the latest version of the PLOS LaTeX template. We have meticulously formatted our manuscript according to the template, ensuring that all packages and formatting instructions are followed precisely. This includes the proper inclusion of references using the plos2015.bst BibTeX style sheet and the incorporation of the .bbl file directly within our .tex document, as instructed.

We believe that these adjustments will greatly enhance the compatibility and readability of our manuscript within the PLOS system. We are confident that our submission now fully complies with the journal's formatting requirements and look forward to the next steps in the review process.

**Comment 5:** Please provide a complete Data Availability Statement in the submission form, ensuring you include all necessary access information or a reason for why you are unable to make your data freely accessible. If your research concerns only data provided within your submission, please write "All data are in the manuscript and/or supporting information files" as your Data Availability Statement.

**Response 5:** In response to your feedback, we have carefully revised our Data Availability Statement to include all necessary access information. Our study solely relies on the data presented within the manuscript and/or supporting information files. Therefore, in accordance with your instruction, we have specified in the statement: "All data are within the manuscript and/or supporting information files."

We understand the essential role of data sharing in scientific research and are committed to adhering to the highest standards of data availability and transparency. By including this explicit statement, we aim to eliminate any ambiguity regarding data access and to facilitate the verification and extension of our findings by the scientific community.

**Comment 6:** We note that Figures 1, 3 and 5 your submission contain [map/satellite] images which may be copyrighted. All PLOS content is published under the Creative Commons Attribution License (CC BY 4.0), which means that the manuscript, images, and Supporting Information files will be freely available online, and any third party is permitted to access, download, copy, distribute, and use these materials in any way, even commercially, with proper attribution. For these reasons, we cannot publish previously copyrighted maps or satellite images created using proprietary data, such as Google software (Google Maps, Street View, and Earth). For more information, see our copyright guidelines:http://journals.plos.org/plosone/s/licenses-and-copyright.

We require you to either (1) present written permission from the copyright holder to publish these figures specifically under the CC BY 4.0 license, or (2) remove the figures from your submission:

1. You may seek permission from the original copyright holder of Figures 1, 3 and 5 to publish the content specifically under the CC BY 4.0 license.

We recommend that you contact the original copyright holder with the Content Permission Form ([http://journals.plos.org/plosone/s/file?id=7c09/content-permission-form.pdf](http://journals.plos.org/plosone/s/file?id=7c09/content-permission-form.pdf)) and the following text:

“I request permission for the open-access journal PLOS ONE to publish XXX under the Creative Commons Attribution License (CCAL) CC BY 4.0 ([http://creativecommons.org/licenses/by/4.0/](http://creativecommons.org/licenses/by/4.0/)). Please be aware that this license allows unrestricted use and distribution, even commercially, by third parties. Please reply and provide explicit written permission to publish XXX under a CC BY license and complete the attached form.”

Please upload the completed Content Permission Form or other proof of granted permissions as an "Other" file with your submission.

In the figure caption of the copyrighted figure, please include the following text: “Reprinted from [ref] under a CC BY license, with permission from [name of publisher], original copyright [original copyright year].”

If you are unable to obtain permission from the original copyright holder to publish these figures under the CC BY 4.0 license or if the copyright holder’s requirements are incompatible with the CC BY 4.0 license, please either i) remove the figure or ii) supply a replacement figure that complies with the CC BY 4.0 license. Please check copyright information on all replacement figures and update the figure caption with source information. If applicable, please specify in the figure caption text when a figure is similar but not identical to the original image and is therefore for illustrative purposes only.

The following resources for replacing copyrighted map figures may be helpful: USGS National Map Viewer (public domain):[http://viewer.nationalmap.gov/viewer/](http://viewer.nationalmap.gov/viewer/). The Gateway to Astronaut Photography of Earth (public domain):[http://eol.jsc.nasa.gov/sseop/clickmap/](http://eol.jsc.nasa.gov/sseop/clickmap/)Maps at the CIA (public domain):[https://www.cia.gov/library/publications/the-world-factbook/index.html](https://www.cia.gov/library/publications/the-world-factbook/index.html) and [https://www.cia.gov/library/publications/cia-maps-publications/index.html](https://www.cia.gov/library/publications/cia-maps-publications/index.html). NASA Earth Observatory (public domain):[http://earthobservatory.nasa.gov/](http://earthobservatory.nasa.gov/).Landsat:[http://landsat.visibleearth.nasa.gov/](http://landsat.visibleearth.nasa.gov/).USGS EROS (Earth Resources Observatory and Science (EROS) Center) (public domain):[http://eros.usgs.gov/#](http://eros.usgs.gov/).Natural Earth (public domain):[http://www.naturalearthdata.com/](http://www.naturalearthdata.com/)

**Response 6:** Thank you for your thorough review and valuable feedback regarding the copyright issues related to Fig 1, 3, and 5 in our submission. We fully appreciate the importance of adhering to copyright guidelines, especially given PLOS ONE's commitment to publishing content under the Creative Commons Attribution License (CC BY 4.0).

Figure Caption Updates: If permissions are granted, we will update the figure captions of the copyrighted figures to include the required text: "Reprinted from [ref] under a CC BY license, with permission from [name of publisher], original copyright [original copyright year]." If replacement figures are used, we will specify in the caption text when a figure is similar but not identical to the original image and is therefore for illustrative purposes only.

**Comment 7:** Please include captions for your Supporting Information files at the end of your manuscript, and update any in-text citations to match accordingly. Please see our Supporting Information guidelines for more information:

http://journals.plos.org/plosone/s/supporting-information.

**Response 7:** In response to your instruction, we have carefully reviewed the Supporting Information guidelines available at the provided link: http://journals.plos.org/plosone/s/supporting-information. We have ensured that all captions for the Supporting Information files are now included at the end of the manuscript. Furthermore, we have meticulously updated any in-text citations to match the newly added captions accordingly.

To adhere to the guidelines, we have named our supporting information files according to the recommended format, including an "S" followed by a number and a descriptive title. We have also ensured that the names of the files match the captions within the manuscript, and each caption includes the supporting information name and number, along with a recommended one-line title for clarity.

We have taken care to maintain the required format for the captions as specified in the guidelines, and we have listed them in a manner that facilitates easy reader access. Additionally, we have verified that the hyperlinks in the published article will correctly direct readers to the corresponding supporting information files.

We believe that these revisions address your concerns and enhance the overall quality and accessibility of our manuscript.

**Review Comments to the Author**

Please use the space provided to explain your answers to the questions above. You may also include additional comments for the author, including concerns about dual publication, research ethics, or publication ethics. (Please upload your review as an attachment if it exceeds 20,000 characters)

**Reviewer #1**:

**Comment 1:** This paper uses online data to calculate water yield WY for Gansu Province in China by subtracting actual evapotranspiration AET from precipitation for each point in a raster. It then looks at temporal and spatial trends in WYs and uses Geodetector analysis and Geographically weighted regression to quantify the influence of natural and anthropogenic factors. Natural factors were found to predominate, especially precipitation and evapotranspiration, but this conclusion provides no insight because WY is calculated directly from AET and precipitation. The paper could be of general interest if the input data and GIS or computer code were made available, but they are not, which is inconsistent with the aim of PONE to promote open science and reproducibility. Specifically, the manuscript does not adhere to PONE criteria #7: "The article adheres to appropriate reporting guidelines and community standards for data availability." As a result, the paper would only be useful as a report for land use plann.

**Response 1:** We fully acknowledge the importance of adhering to PONE's criteria for data availability and promoting open science and reproducibility. We understand your concern regarding the calculation of WY and the subsequent analysis of natural and anthropogenic factors. You have correctly pointed out that our conclusion about the predominance of natural factors, particularly precipitation and evapotranspiration, might seem obvious given that WY is calculated directly from these variables. To address this issue, we would like to emphasize that our study aimed to investigate the spatial and temporal trends in WY and to quantify the influence of various factors using Geodetector analysis and Geographically weighted regression. While it is true that WY is calculated from precipitation and evapotranspiration, our analysis goes beyond this simple calculation by exploring the complex interactions and relationships among these variables and other potential factors.

Regarding the data availability, we recognize the significance of making our input data and GIS or computer code accessible to other researchers. We are committed to promoting open science and reproducibility and will make every effort to provide these resources upon acceptance of our manuscript. We will ensure that all necessary data and code are deposited in an appropriate repository and will include a detailed Data Availability Statement in our revised manuscript.

We believe that our study has the potential to contribute to the broader scientific community, beyond its immediate application for land use planning. Thank you once again for your valuable feedback and constructive criticism. We are confident that our revised manuscript, with improved clarity and adherence to PONE's criteria for data availability, will meet the expectations for publication.

**Comment 2:** Describe where the data may be found in full sentences: "This text is appropriate if the data are owned by a third party and authors do not have permission to share the data."

This sounds like a copout and is inconsistent with PONE policy. Who is the third party? Based on Table 1 it looks like all of the data they used is publicly available. The authors should therefore compile the data and publish it in an online repository so that reviewers and readers have the opportunity to verify their analysis.

Problem: I tried the url's in Table 1 for precipitation and evapotranspiration that are used to calculate water yields and got the error "This www.geodata.cn page can’t be found".

If a GIS was used, then it should be made available online. If code was written to analyze the data, then it should also be made available.

**Response 2:** Thank you for your detailed evaluation of our manuscript and for highlighting the crucial aspects related to data availability and accessibility. We appreciate your insights and the time you have taken to review our work.

We understand your concerns regarding the statement about data ownership by a third party, as it may have given the impression of a lack of transparency or adherence to PONE's policy on open science and reproducibility. We want to clarify that this statement was not intended as a copout but rather as an acknowledgment of potential limitations that could arise from data access restrictions. However, upon further reflection and in light of your comments, we recognize that all the data used in our study are indeed publicly available, as indicated in Table 1.

We deeply regret the inconvenience caused by the issue with the URLs provided in Table 1 for precipitation and evapotranspiration data. We acknowledge that the error message "This www.geodata.cn page can’t be found" prevents access to these data, which is critical for the verification and reproducibility of our analysis. To address this, we will carefully review and update the URLs to ensure they direct to the correct and accessible data sources. **(Line number 168).**

We sincerely apologize for any confusion or inconvenience our previous handling of data availability may have caused. We are fully committed to adhering to PONE's policies and promoting open science. We are confident that by addressing these concerns, our revised manuscript will meet the expectations for publication and contribute positively to the scientific community.

**Comment 3:** Lines 20-22: "The results show that the WYs in Gansu Province fluctuated between 278.37 and 381.96 mm during .

2000–2022, with an average WY of 61.09 mm." How can the average lie outside the range? OK, lines 218-222 show that the larger numbers are min and max precipitation. So the sentence should read the same as lines 222-224: "

The minimum value of WYs appeared in 2015 (38.85 mm), the maximum value appeared in 2021 (114.11 mm), and the multiyear average value was 61.09 mm."

**Response 3:** You correctly pointed out the inconsistency in our statement regarding the water yields (WYs) in Gansu Province. Specifically, we acknowledged that the WYs fluctuated between 278.37 and 381.96 mm during 2000-2022 but erroneously reported an average WY of 61.09 mm, which clearly lies outside the given range. Upon reviewing our data and analysis, we understand now that the larger numbers mentioned in lines 218-222 represent the minimum and maximum precipitation values, not the WYs themselves.

We deeply regret this error and acknowledge that it could mislead readers and undermine the credibility of our findings. To rectify this, we will revise the sentence in question to align with the correct data presented in lines 222-224. The revised sentence will read: "The minimum value of WYs appeared in 2015 (38.85 mm), the maximum value appeared in 2021 (114.11 mm), and the multiyear average value was 61.09 mm. We sincerely apologize for any confusion this error may have caused and appreciate your constructive criticism in helping us improve the accuracy and clarity of our manuscript. We are committed to ensuring that all data and findings are presented accurately and transparently. **(Line number 246-248).**

**Comment 4:** You need to define water yield at the beginning of the Intro like you do in lines 147-8.

**Response 4:** Regarding your comment on line 69, we acknowledge that our use of terminology may have led to some confusion. You are correct in pointing out that WYs themselves are not processes, but rather are the results of complex ecohydrological processes such as precipitation, runoff, infiltration, and evapotranspiration (ET). To clarify this, we will revise the text to state: "WYs are determined by complex ecohydrological processes, including precipitation, runoff, infiltration, and ET." Additionally, we will incorporate the equation you provided, WY = precipitation - ET - infiltration - water usage + runoff, to further elucidate the relationship between these processes and water yield. **(Line** **number 40-50).**

Furthermore, in response to your suggestion to define water yield at the beginning of the Introduction, we agree that this would improve the accessibility of our manuscript to a broader audience. We will therefore include a clear and concise definition of water yield, similar to the one provided on lines 147-8, early in the Introduction to establish a solid foundation for the subsequent discussions.

**Comment 5:** Line 69: Here and elsewhere you state "WYs are complex ecohydrological processes". WYs are not processes; rather, processes like precipitation, runoff, infiltration and evapotranspiration ET determine the water yield: WY = precipitation - ET - infiltration - water usage + runoff

**Response 5:** Regarding your comment on Line 69, where and elsewhere we stated that "WYs are complex ecohydrological processes," we understand your concern. You are correct in pointing out that WYs themselves are not processes but rather a time frame used in hydrological studies. Processes such as precipitation, runoff, infiltration, and evapotranspiration are indeed the ones that determine the characteristics and dynamics of WYs. In response to your suggestion, we have revised the manuscript to clarify this point. Specifically, we have modified the relevant sentences to accurately reflect that WYs are a time period during which these ecohydrological processes occur, rather than the processes themselves. **(Line number 37-52).**

**Comment 6:** Line 251: "with a linear trend of 2.21 mm/yr at the raster scale." Do you mean that the average trend for Gansu province was 2.21 mm/yr? Here and other places you should replace "raster scale" with "Gansu province".

**Response 6:** Thank you very much for your meticulous review of our paper and your valuable comments. In response to your concern regarding the statement on Line 251, "with a linear trend of 2.21 mm/yr at the raster scale," we deeply appreciate your insight and have given it careful consideration. You correctly pointed out that the use of "raster scale" in this context may cause confusion, as it does not clearly convey the specific geographical area of our analysis.

Following your suggestion, we have revised the text to replace "raster scale" with "Gansu province," thereby more accurately indicating that the linear trend of 2.21 mm/yr pertains to this particular region. This modification not only enhances the clarity of the statement but also ensures the accuracy of the information, facilitating a better understanding of our research findings by the readers. **(Line number 275-276, 280 and 285).**

**Comment 7:** Section 3.3 Analysis of WYs drivers: "the main influences on the WYs in the study area were of precipitation (0.49), DEM (0.25), slope (0.23), temperature (0.16), and evapotranspiration (0.14). The one-way probe results for 2010 were approximately identical to those for 2000. However, in 2022, precipitation and evapotranspiration began to dominate." Since WY is calculated from precipitation and evapotranspiration, I don't think it's meaningful to use a statistical method to quantify their influence.

**Response 7:** Firstly, I would like to express my sincere gratitude to the reviewers for their invaluable comments. WYs typically refer to the residual water volume after subtracting evapotranspiration and other losses from precipitation, representing the actual water that flows into rivers or recharges groundwater. The reviewer might argue that since WYs is already calculated based on these two factors, analyzing their impacts using statistical methods would be redundant or meaningless, as WYs are inherently a function of these two variables. However, statistical methods are not only employed to examine the interactions between precipitation and evapotranspiration but also to investigate whether their influences on WYs are consistent across different temporal and spatial scales. Moreover, there may be other variables or factors that warrant consideration, such as topography, socio-economic activities, and land-use changes, which can indirectly affect WYs by influencing evapotranspiration or the redistribution of precipitation. Statistical methods can help uncover dynamic changes, nonlinear relationships, or sensitivities under varying conditions among these variables. Furthermore, during our initial literature review and analysis, the correlation between WYs and precipitation and evapotranspiration was not absolute. For instance, Wu et al. (2024) found that the primary factors influencing WYs were precipitation and land use, while the results of Ma et al. (2024) indicated that precipitation and distance to government were the main determinants of WYs. Therefore, precipitation and evapotranspiration are not necessarily the decisive factors affecting WYs. The impacts of these influencing factors on WYs vary across different times and spaces, and the interactions between precipitation, evapotranspiration, and other factors also exert an influence on WYs. Consequently, specific research findings still require statistical analysis methods to provide intuitive evidence.

**References**

Wu L, Luo Y, Pang S, et al., 2024. Factor analysis of hydrologic services in water-controlled grassland ecosystems by InVEST model and geodetector. *Environmental Science and Pollution Research.* 31(13): 20409-20433.

Ma X, Liu S, Guo L, et al., 2024. Evolution and Analysis of Water Yield under the Change of Land Use and Climate Change Based on the PLUS-InVEST Model: A Case Study of the Yellow River Basin in Henan Province. *Water.* 16(17): 2551.

**Comment 8:** Fig. 4: You have three plots with identical x and y categorical axes, but in the caption you don't explain what is different between plots a), b), and c). Are they for different years? Same problem for Figure 5 - I think the captions should say "a) year 2000; b) year 2010; c) year 2022".

**Response 8:** Thank you very much to the reviewer for the comments regarding the graphical representation. We have taken the feedback into consideration and made corresponding adjustments to **Fig. 4** by adding the respective years to each of the three plots. The specific modifications are illustrated in the revised figure. Additionally, for **Fig. 5**, we have annotated each driving factor column with the corresponding year and included a note in the figure caption for clarification. **(Line number 316-317).**

|  |  |
| --- | --- |
|  |  |

**Fig. 4.** Interaction between drivers affecting water production and one-way detection results, 2000-2022.

**Comment 9:** Lines 307-9: "The DEM was positively correlated with WYs throughout the region, with the strength increasing gradually from the north to the south." Do you mean elevations, or slopes? It's meaningless to say that a digital elevation model is correlated with water yield. I think you must mean elevation.

**Response 9:** We are deeply grateful to the reviewer for their meticulous attention to the precision of our terminology. We have taken note of the improper use of "DEM" in our manuscript. Digital Elevation Model, abbreviated as DEM, is a digital representation of ground topography through a limited set of terrain elevation data. It should not be used interchangeably to simply denote elevation. In light of this, we have replaced the inappropriate words in Lines 307-309 accordingly. **(Line number 369).**

**Comment 10:** I don't think the geodetector results add anything to the paper. The GWR model is easier to understand and "is highly explanatory of WYs".

**Response 10:** We appreciate the questions raised by the reviewers. This issue was carefully considered during the initial design phase of our article. Both the Geodetector and the Geographically Weighted Regression (GWR) model possess the capability to explain the impact of driving factors on water yield. However, these two models exhibit distinct advantages. The Geodetector constitutes a set of statistical methods for detecting spatial heterogeneity and unveiling the underlying driving forces. This method not only ensures immunity to multicollinearity among independent variables but also captures the influence of interactions between variables on water yield. These findings contribute to a deeper understanding of how driving factors affect water yield. On the other hand, the GWR model provides insights into the varying strengths of different driving factors' influences on water yield across spatial scales. Collectively, the results from these two models are complementary and conducive to a profound exploration of the driving mechanisms underlying water yield.

**Comment 11:** Lines 322-323: "The WY value is the difference between the average annual precipitation and AET on a raster scale based on the principle of water balance." This should be stated at the beginning of the Introduction.

**Response 11:** We appreciate your suggestion to clarify the concept of the WY value earlier in the Introduction. In response to your comment, we agree that introducing the WY value-defined as the difference between the average annual precipitation and AET on a raster scale based on the principle of water balance-at the outset of the Introduction will enhance the reader's understanding and provide a clearer context for the subsequent discussions. We will revise the manuscript accordingly to ensure that this fundamental definition is presented upfront, thereby facilitating a smoother flow of information and improving the overall coherence of the paper. **(****Line number 37-52).**

**Comment 12:** Lines 328-330: "The capability of precipitation to reach the surface and be converted into runoff is constrained by different land-use types." What about infiltration?

**Response 12:** We would like to express our gratitude to the reviewer for highlighting this critical issue in their feedback. It is acknowledged that the infiltration process is a pivotal component in the conversion of precipitation into surface runoff. The land use types, including forest land, crop land, and built up land, exert a direct influence on the mechanism of runoff generation by modulating soil infiltration capacity. While the present study did take into account the role of infiltration in its analysis, the association with land use may not have been fully elucidated in the original text. Consequently, we have made modifications to this section in the original text. **(****Line number 431-437).**

**Comment 13:** Since WY is calculated using ET, it would be valuable to explain how ET was measured.

**Response 13:** You are absolutely right in pointing out that since WY is calculated using ET, it is crucial to provide a clear explanation of how ET was measured. We appreciate your emphasis on the importance of transparency and rigor in methodological details. In response to your suggestion, we will include a detailed description of the methodology used to measure ET in the revised manuscript. Specifically, ET based on the Penman-Monteith formula was interpolated into the grid data using ANUSPLINE software. We believe that this addition will enhance the clarity and reproducibility of our study, and we are grateful for your guidance in improving the quality of our work. Thank you once again for your valuable feedback. **(Line number 168).**

**Comment 14:** I don't think it was stated anywhere what the spatial resolution of the analysis was. What is the pixel size in the raster?

**Response 14:** In response to the reviewer's comments, we have already addressed in Section 2.2, Data requirement and preparation, that all data were uniformly resampled to 30 meters using the nearest neighbor interpolation method within the ArcGIS 10.7 software. **(Line number 147-167).**

**Comment 15:** Lines 337-340: "the forest land in Gansu Province is mainly distributed in the southwestern mountainous area with a relatively high elevation, and the warm and humid airflow is compelled to lift by the topography to straightforwardly form the terrain rain." I think you are describing orographic precipitation. What is the predominant wind direction in this area?

**Response 15:** We concur with the issue you mentioned in lines 337-340 regarding the distribution of forest land and the mechanism of terrain rainfall formation in Gansu Province. The assertion that terrain rainfall in the region, that is to say, rainfall formed by terrain uplift, is the dominant factor, is indeed valid. This is a point that was not fully elucidated in our research.

In response to the inquiry regarding the predominant wind direction in the region, our analysis of pertinent meteorological data indicates that the dominant wind direction in the southwestern mountainous areas of Gansu Province, particularly in the Longnan Mountains and Gannan Plateau regions, is significantly influenced by seasonal and topographical variations. During summer months, the prevailing southeast monsoon influences the region, resulting in the influx of warm and humid airflow from the southeast. The interaction of these airflows with the region's imposing topography results in the formation of abundant rainfall, characterized by a decrease in temperature and condensation of water vapor. Conversely, during winter, the influence of the northwest monsoon is more pronounced. However, the relatively dry airflow and lower temperatures characteristic of this season result in comparatively less rainfall. However, on average throughout the year, especially in summer, the southeast monsoon is the predominant wind direction that forms topographic rainfall in the region. **(Line number 359-371).**

In the event of a revision of the paper, the information regarding the impact of terrain rainfall on the distribution of forests in the southwestern mountainous areas of Gansu Province will be explicitly supplemented. Furthermore, the interaction of this climate characteristic with the local forest ecosystem will be elaborated upon. We would like to express our gratitude once more for your insightful feedback. Your feedback is instrumental in enhancing the quality of our research.

**Comment 16:** Section 4.2 is one extremely long paragraph. Break this up to make it more readable.

**Response 16:** We have reorganized the content of Section 4.2 in response to the reviewer's comments. The first paragraph primarily discusses the results obtained from the Geodetector analysis. The second paragraph focuses on the influence of natural factors on WYs under the GWR model. The third paragraph mainly explores the impact of anthropogenic factors on WYs.**(Line number 395-446).**

**Reviewer #2**: The work is like a case study Why the Global readers should show interest in your paper?

Please add generic framework

**Response:** Thank you for your insightful comment, which prompts us to further elucidate why our work should be of interest to a global audience. Our paper presents a comprehensive case study that delves into the dynamic changes and driving mechanisms of water yield services in arid and semi-arid regions, an area of universal relevance and significance.

The generic framework we employ in our analysis is designed to be both robust and adaptable, enabling its application across diverse contexts and settings. By examining the natural and human-driven factors influencing water production services in arid and semi-arid regions, it helps establish a realistic connection between ecological management practices and maximizing ecosystem service benefits, thereby contributing to addressing extreme climate change. This framework not only enhances the generalizability of our findings but also ensures their relevance to a wide range of stakeholders, including policymakers, practitioners, and scholars worldwide.

**Comment 1:** What is the take home message of the study? is it just application of InVest model? also to a different province? How the results will change if another model is used? How will you deal with the uncertainty?

**Response 1:** In response to the reviewer's comments, the take-home message of our study extends beyond the mere application of the InVest model to a different province. While the InVest model served as a valuable tool for assessing ecosystem services in the study area, our research contributes more broadly by demonstrating the feasibility and adaptability of such models in diverse geographical contexts. The key finding is that, with appropriate calibration and local data integration, the InVest model can provide insightful estimates of ecosystem service values, which can inform policy and management decisions.

Regarding the use of another model, it is acknowledged that different models may yield varying results due to differences in underlying assumptions, algorithms, and input data requirements. However, the robustness of our findings lies in the consistent patterns observed across the key ecosystem services assessed, which suggests that the general conclusions are likely to hold even if another model were used. Nevertheless, future research could benefit from a comparative analysis of multiple models to further explore these potential differences and their implications.

As for dealing with uncertainty, we recognize that all models, including the InVest model, are subject to uncertainties arising from data limitations, model assumptions, and the inherent complexity of ecosystems. To address this, we employed a range of sensitivity analyses and uncertainty assessments to evaluate the robustness of our results. These analyses helped us to identify the key sources of uncertainty and to assess the potential impact on our conclusions. Furthermore, we emphasize the importance of transparent reporting of model assumptions, data sources, and uncertainty estimates to facilitate critical evaluation and informed decision-making.

In summary, our study not only applies the InVest model to a new province but also contributes to the broader understanding of ecosystem service modeling and its application in diverse contexts. We acknowledge the potential for varying results with different models and have taken steps to address uncertainty in our analysis. We hope that our research will stimulate further investigation into the use of ecosystem service models for sustainable land management and conservation planning.

**Comment 2:** Fig. 5. Spatial distribution of regression coefficients for drivers affecting WYs- legends are not visible ; please revise.

**Response 2:** In response to the reviewer's comments, we have regenerated a high-resolution composite image (Fig. 5). Due to the nature of this composite image, it was not feasible to maintain the size of the individual components while simultaneously ensuring clear visibility of the legend. **(Line number 341).**

**Fig. 5.** Spatial distribution of regression coefficients for drivers affecting WYs, 2000–2022.

**Comment 2:**L80 ………Li, 2022; Liu et al.).- complete it.

**Response 2:** In response to the reviewer's comment on line 80, we have ensured that the citation is complete. We apologize for any oversight in the initial submission and appreciate the reviewer's attention to detail. We have carefully reviewed the manuscript to ensure that all citations are accurate and complete, adhering to the academic standards for referencing.

**Comment 3:** the spatial non-stationarity of parameters-what do you mean by this ? heterogeneity?

**Response 3:** Spatial non-stationarity in the parameters of the Geographically Weighted Regression (GWR) model refers to the phenomenon whereby the regression coefficients (parameters) of the independent variables in the model vary across geographical locations. Unlike traditional global regression models, such as Ordinary Least Squares (OLS) regression, GWR permits each geographical unit (e.g., region, point) to have its own unique regression coefficients, thereby capturing the spatial heterogeneity in the relationships among variables. Consequently, spatial non-stationarity of the parameters can also be described as spatial heterogeneity.

**Comment 4:** Actual et etc AET ?? use proper and consistent notations.

**Response 4:** Regarding your comment on the notation 'Actual et etc AET??', we fully appreciate your emphasis on the importance of using proper and consistent notations in academic writing. We acknowledge that there may have been some inconsistencies in our use of 'Actual', 'etc.', and the potential abbreviation 'AET' in the manuscript. To enhance the academic rigor and readability of the paper, we will thoroughly review the entire text to ensure that all symbols, abbreviations, and terms are used consistently and in accordance with academic conventions. As for the specific abbreviation 'AET', we will either clarify its full name and specific meaning in the context of the paper or replace it with a more common and clear expression. Thank you again for your careful guidance. We will make the necessary revisions to submit a more polished and standardized manuscript.
